# Evaluating the Diagnostic Potentials of Circulating Tumor DNA against Melanoma: A Systematic Review and Meta-Analysis

**DOI:** 10.1155/2022/6233904

**Published:** 2022-10-12

**Authors:** Jianchao Zhang, Da Qian, Xiaochen Xu, Ming Xu, Ke Wang, Hui Lu, Guoliang Shen

**Affiliations:** ^1^Department of Burn and Plastic Surgery-Hand Surgery, First People's Hospital of Changshu City, Changshu Hospital Affiliated to Soochow University, No. 1 Shuyuan Street, Changshu, Soochow 215500, China; ^2^Department of Burn and Plastic Surgery, First Affiliated Hospital of Soochow University, No. 188 Shizi Street, Soochow 215000, China

## Abstract

**Background:**

The accurate detection of circulating tumor (ct) DNA is affected by multiple factors, and several controversies still persists regarding clinical applications. In order to assess the consistency of ctDNA gene mutation detection findings in matched melanoma tissue samples and peripheral blood, a meta-analysis was performed and provided evidence-based analysis for its clinical applications.

**Method:**

As of May 20, 2019, the database has been searched using the Embase, PubMed, and Cochrane Library search engines. The ctDNA investigations mentioned in this review may be used to directly or indirectly get the true positive (TP), true negative (TN), false positive (FP), and false negative (FN) values of melanoma patients. To be excluded from the study are duplicate publications, research that do not offer a full text, inadequate material or an inability to extract data, and animal trials.

**Results:**

Overall, the pooled specificity, sensitivity, NLR, PLR, and DOR were 0.94 (95% CI: 0.91-0.96), 0.73 (95% CI: 0.70-0.75), 0.32 (95% CI: 0.22-0.45), 8.21 (95% CI: 4.67-14.43), and 32.72 (95% CI: 14.81-72.30), respectively. Additionally, we calculated AUC by drawing the SROC curve, and the value of AUC is 0.9287, which indicates that the accuracy of ctDNA in diagnosing melanoma is 92.87% of the gold standard. Furthermore, we conducted a subgroup analysis for different countries, sample sources, and ctDNA detection methods. The pooled results showed that different countries, sample sources, and ctDNA detection methods showed significantly large differences in terms of sensitivity of ctDNA in diagnosing melanoma, while the specificity basically remained the same.

**Conclusion:**

We discovered that the diagnostic outcomes between matched tumor samples and ctDNA remained more reliable in melanoma patients. ctDNA has the advantages of low trauma, convenient dynamic monitoring, and simple operation. ctDNA is expected to become an auxiliary method for the diagnosis of melanoma gene mutations.

## 1. Introduction

Melanoma is a very aggressive skin tumor caused by the excessive proliferation of melanocytes. It mostly occurs in the skin, mucous membrane, and extremities. Although its incidence is only 10% of skin tumors, it is related to 80% of skin tumor deaths [1]. The 2018 Global Cancer Report indicated that there were 287,723 new cases of melanoma and 60,712 deaths [2]. In the early stage of melanoma, surgical resection is the first choice, while for advanced patients, traditional radiotherapy and chemotherapy showed very little effects for melanoma patients who cannot be surgically removed or who have metastasized and have BRAF V600E mutations. The treatment of melanoma has entered the age of targeted therapy after the U.S. Food and Drug Administration (U.S. Food and Drug Administration, FDA) authorized vemurafenib as a targeted drug in 2011 [3].

Traditionally, archival formalin-fixed, paraffin-embedded (FFPE) tumor tissues obtained after diagnosis and/or additional biopsies or surgery are used to identify somatic mutations. The high risk of puncture, the inability to find the tumor in an anatomical position, the high expense, and the intricacy of the tumor tissue are just a few of the problems with mutation testing on archival tumor material, though [4, 5]. Circulating tumor DNA (ctDNA) detection is an emerging method that has been used to detect genetic mutations in humans in recent years. ctDNA is a DNA fragment that enters the blood circulatory system after the DNA of tumor cells falls off or undergoes apoptosis, which can be used as a special tumor marker. It is possible that ctDNA analysis might provide a more comprehensive view of the tumor's subclones [6]. A larger amount of tumor-specific somatic mutations may be discovered in circulating free DNA (ctDNA) in individuals with advanced cancer than in healthy persons [7, 8]. Pinzani et al. [9] pointed out that in patients with melanoma, the sensitivity of ctDNA detection was 72%, the specificity was 89%, and the consistency with tumor pathology detection results was 80% compared with the results of tumor tissue detection. A report by Tang et al. [10]. Demonstrated that the test results between tumor tissue and ctDNA were 70% consistent in 58 melanoma patients. There is still debate over the relevance of ctDNA detection in clinical settings because its accuracy depends on a number of variables, including the detection tool, sample source, and area.

In this study, quantitative Meta-analysis was used to evaluate the consistency of ctDNA gene mutation detection results in matched melanoma tissue samples and peripheral blood and provide evidence-based basis for clinical application.

## 2. Methods

### 2.1. Inclusion/Exclusion Criteria for Literature

The following were the inclusion criteria: (1) true positive (TP), true negative (TN), false positive (FP), and false negative (FN) values of patients with melanoma may be directly retrieved from the original article or indirectly based on the information supplied in the literature. (2) ctDNA were used for the diagnosis of melanoma in patients with the following exclusionary conditions: (1) FeNO patients' true positive (TP), false positive (FP), true negative (TN), and false negative (FN) diagnostic values are not included in the study data and cannot be estimated; (2) studies lacking full text, inadequate information, or the incapacity to extract data; (3) case reports, reviews, and systematic reviews. (4) Repeated publishing.

### 2.2. Search Strategy

We searched Pubmed, Embase, and the Cochrane Library from the time the databases were first launched until May 2021 for the purposes of this meta-analysis. The following are the mesh glossary terms: “Circulating Tumor DNA,” “Cell-Free Tumor DNA,” and “Melanoma”.

### 2.3. Literature Screening and Data Extraction

The literature review, screening, and data extraction are carried out independently by two researchers. Disagreements are settled through discussion or by asking a third party for their opinion. An author's name and year, as well as a sample's size and origin, as well as the technique used to identify ctDNA and TP, FP, TN, and FN may all be found in the data that is extracted and used to diagnose patients with melanoma.

### 2.4. Literature Quality Assessment

Two researchers used the QUADAS-2 tool [11] to assess the quality of each piece of included literature, which consists of 11 different components (for details, see the labelling of the bias risk graph and the bias risk summary graph in the results section). After cross-checking the findings, if there are still differences of opinion, a decision will be reached via discussion or consultation with a third party based on the assessment results being classified as “high” or “low” risk. Review manager 5.3 software is used to build bias risk maps and bias risk summary maps once all items have been evaluated.

### 2.5. Data Synthesis and Statistical Analysis

Using Meta-Disc1.4 software, which also did heterogeneity testing, the sensitivity, specificity, and 95 percent confidence interval of each independent research were calculated. First, the Spearman correlation coefficient *P* value is determined. This is done if the correlation coefficient *P* value falls below 0.05, which indicates a threshold effect. The area under the SROC curve (AUC) is determined. The opposite is true, which suggests that there is no threshold effect. In this case, we may combine the sensitivity, specificity, positive likelihood ratio (PLR), negative likelihood ratio (NLR), and diagnostic odds ratio (DOR) as well as other indications and construct a complete SROC curve. The heterogeneity test caused by nonthreshold effects is calculated by calculating the *χ*2 or *Q* value and the I2 value. Random effects models are utilized if *I*^2^ is more than 50%; otherwise, the fixed effects models are used. Heterogeneity may be tracked down via sensitivity analysis or subgroup analysis. If the heterogeneity still exists, a random effects model is used; otherwise, a descriptive analysis is used instead. Deeks' funnel plot asymmetry test may be performed using STATA15.1 software to determine publication bias.

## 3. Results

### 3.1. The Results of Literature Search

**Figure 1:**
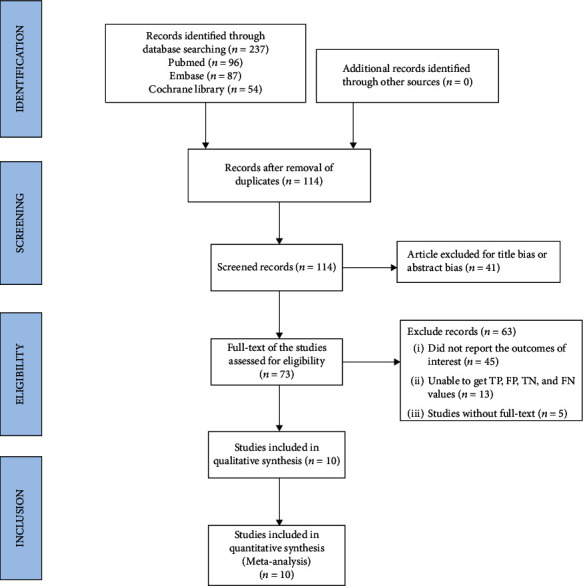
Showing the flowchart for selected studies.

A total of 237 studies were selected from the database for this research. 114 studies remained after removing duplicates. After going through the titles and abstracts, 73 papers were found. It was eventually completed by going through the full-texts of the 10 studies (Figure 1).

### 3.2. Baseline Characteristics and Study Quality

#### 3.2.1. Baseline Characteristics

Additionally, Table 1 displays the baseline characteristics and quality rating of the included studies. 10 publications with 1430 patients were included in this meta-analysis. There are 4 articles from Europe and 6 articles from North America. Most of the literature has been published in the past 5 years; the research objects are mainly patients with stage III to IV; ctDNA detection methods were mainly allele-specific polymerase chain reaction (Allele-specific polymerase chain reaction, allele-specific PCR), BEAMing, UltraSEEK, etc.

#### 3.2.2. Quality Assessment of the Included Studies

**Figure 2:**
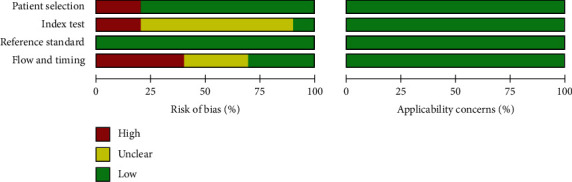
A graph of methodological quality.

**Figure 3:**
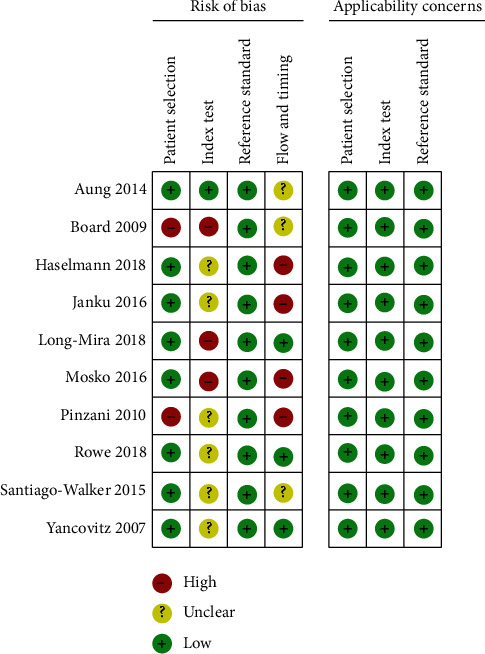
An overview of the methodological quality.

We assessed the methodological quality of each study in accordance with the QUADAS-2 criteria from four angles. Six of the trials to be reviewed did not mention the use of testing blinding, and the other three studies were carried out knowing the outcomes of the tissue test (Figure 2), so there may be unknown or significant risk variations. Since not all patients were included, or there was an inappropriate time interval between the study to be evaluated and the gold standard, the risk of deviation of the case flow and progress of 8 studies was unknown or high. In all studies, the applicability is very high (Figure 3).

### 3.3. Results of Meta-Analysis

**Figure 4:**
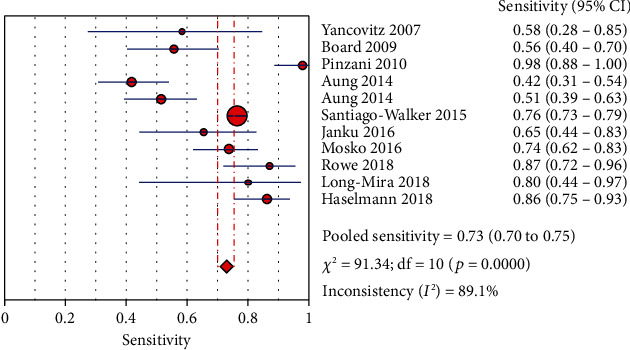
The sensitivity of ctDNA in diagnosing melanoma.

**Figure 5:**
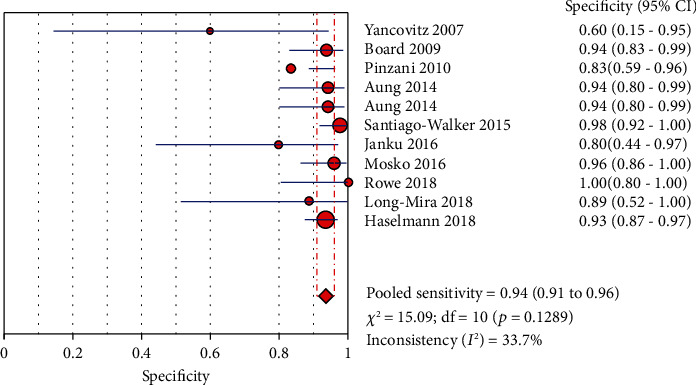
The specificity of ctDNA in diagnosing melanoma.

**Figure 6:**
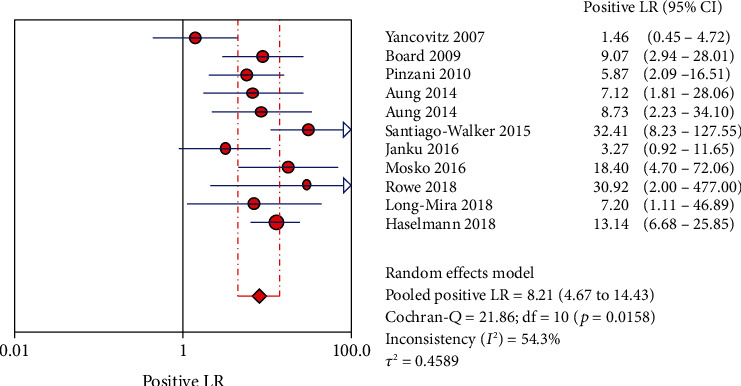
The PLR of ctDNA in diagnosing melanoma.

**Figure 7:**
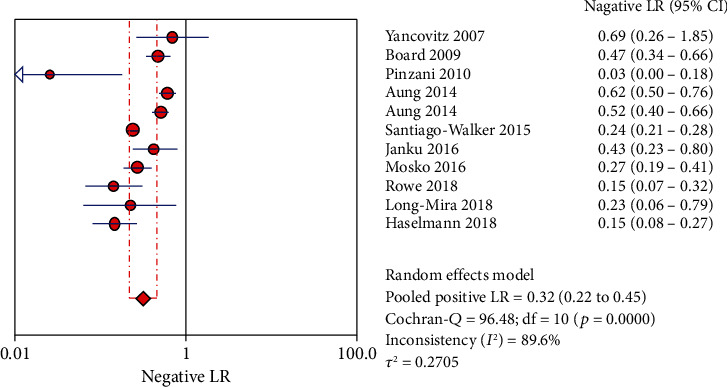
The NLR of ctDNA in diagnosing melanoma.

**Figure 8:**
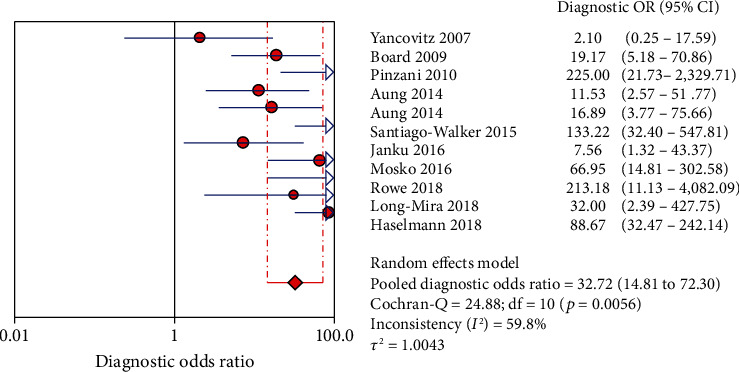
The DOR of ctDNA in diagnosing melanoma.

**Figure 9:**
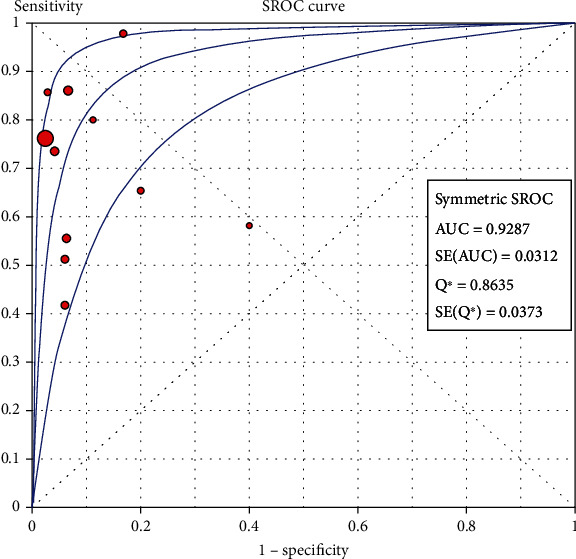
The SROC curve of ctDNA in diagnosing melanoma.

The Spearman correlation value is -0.564 (*P* = 0.090 > 0.05), suggesting the absence of a threshold effect. The combined sensitivity of *χ*2 = 91.34 (*P* ≤ 0.001) and *I*^2^ = 89.1 percent indicates that there is heterogeneity produced by nonthreshold effects, hence the random effects model is employed to combine sensitivity. The combined specificity of *χ*2 = 15.09 (*P* = 0.1289), *I*^2^ = 33.7 percent, indicates that there is no heterogeneity produced by nonthreshold effects, hence the fixed effects model is employed to combine the specificity. There is heterogeneity due to nonthreshold effects in the combined PLR of Cochran-*Q* = 21.86 (*P* = 0.0158), *I*^2^ = 54.3 percent, hence the random effects model is employed to combine the PLR. The random effects model is utilized to combine the NLR because the combined NLR of Cochran-*Q* = 96.48 (*P* ≤ 0.001), *I*^2^ = 89.6 percent, demonstrating heterogeneity driven by nonthreshold effects. Random effects model is used to combine Cochran-combined *Q*'s DOR of 24.88 (*P* = 0.0056) and *I*^2^ of 59.8 percent, which indicates that there is heterogeneity due by nonthreshold effects. The pooled sensitivity, specificity, PLR, NLR, and DOR are 0.73 (95% CI: 0.70-0.75), 0.94 (95% CI: 0.91-0.96), 8.21 (95% CI: 4.67-14.43), 0.32 (95% CI: 0.22-0.45), and 32.72 (95% CI: 14.81-72.30), respectively. Additionally, we calculate AUC by drawing the SROC curve, and the value of AUC is 0.9287, which indicates that the accuracy of ctDNA in diagnosing melanoma is 92.87% of the gold standard (Figures 4–9).

### 3.4. Subgroup Analysis

The research is somewhat heterogeneous, which may be due to various nations, sample sources, and ctDNA detection techniques. For the three aforementioned potential causes, we thus performed a subgroup study. The pooled sensitivity of North America is 0.76 (0.73-0.79), while the pooled sensitivity of Europe is 0.63 (0.57-0.69). The pooled specificity of North America is 0.94 (0.91-0.97), and the pooled specificity of Europe is 0.93 (0.88-0.97). The AUC of ctDNA in European melanoma patients is higher than that in North American patients (0.9576 vs. 0.8018).

In terms of sample source, the pooled sensitivity of plasma is 0.76 (0.73-0.78), while the pooled sensitivity of serum is 0.47 (0.38-0.56). The pooled specificity of plasma is 0.94 (0.91-0.96), and the pooled specificity of serum is 0.94 (0.86-0.98).

In addition, the results of the detection method showed that the sensitivity of Allele-specific PCR detection was 0.60 (0.54-0.65), while the sensitivity of BEAMing detection was 0.78 (0.74-0.81). The pooled specificity of Allele-specific PCR detection was 0.92 (0.86-0.95), and the sensitivity of BEAMing detection was 0.96 (0.92-0.98) (Table 2).

### 3.5. Publication Bias

**Figure 10:**
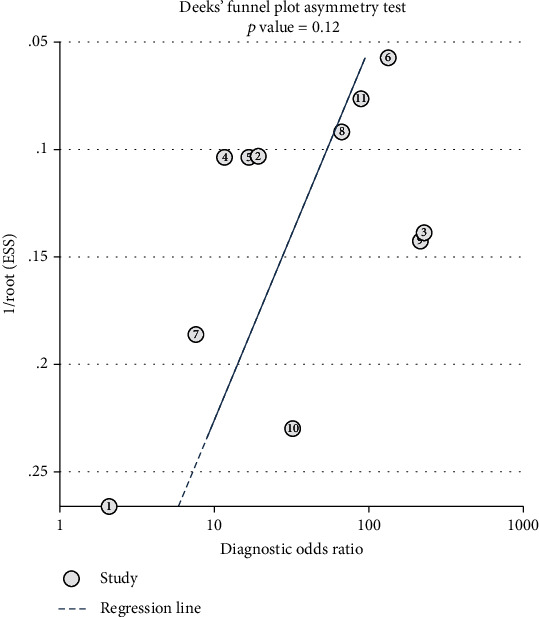
Deek's funnel plot asymmetry test of ctDNA in diagnosing melanoma.

The following is an example of the study's funnel plot. In this research, the *P* value of Deek's funnel plot asymmetry test is 0,12, which indicates that there is no apparent publication bias (Figure 10).

### 3.6. Sensitivity Analysis

Analyzing each included study one at a time to see if single included research has an undue influence on meta-analysis findings is known as a sensitivity analysis. Findings from the meta-analysis indicated no studies had a significant influence on its findings; this suggests the remaining studies' findings are consistent and credible.

## 4. Discussion

The new ctDNA can be utilized as a supplement to tissue biopsy for clinical diagnosis and disease monitoring due to its advantages of noninvasiveness, ease of access, continuous sampling, and overcoming tumor heterogeneity [21]. Traditional tissue biopsy has numerous inherent drawbacks. However, due to the existence of many influencing factors, the diagnostic value of ctDNA in melanoma is still controversial. In this study, 10 articles that met the inclusion criteria were meta-analyzed, and the subjects involved a total of 1430 melanoma patients.

The combined sensitivity of ctDNA for melanoma detection was 0.73 (95% CI: 0.70-0.75), combined specificity was 0.94 (95% CI: 0.91-0.96), and combined AUC was 0.9287. It should be noted that AUC is a comprehensive index, and the closer its value is to 1, the higher the diagnostic value. Our pooled results show that the diagnostic accuracy of ctDNA is 92.87% of the gold standard, indicating that it has a higher diagnostic value in melanoma.

The pooled PLR is 8.21 (95% CI: 4.67-14.43), which indicates that the probability of ctDNA detection in melanoma patients is about 8.21 times that of false positives. The combined NLR is 0.32 (95% CI: 0.22-0.45), indicating that 32% of false negatives may be present in the negative results of ctDNA. DOR is the ratio of the positive results in the experimental group to the positive results in the control group, which can better reflect the “differentiation” ability of the diagnostic test, and the DOR value is positively correlated with its discrimination ability. The pooled DOR is 32.72 (95% CI: 14.81-72.30), indicating that ctDNA detection has a higher comprehensive diagnostic efficiency.

The accuracy of ctDNA detection was examined in this study along with its influencing factors. [13] Board et al. pointed noted that there is a strong correlation between ctDNA level and tumor stage, that ctDNA mutations rely on tumor stage, and that early detection is typically inaccurate. Compared with stage I patients, stage IV patients have higher levels of ctDNA [22], and ctDNA levels are related to tumor metastasis [23]. In addition, the heterogeneity of the tumor may result in inconsistent gene mutation detection results between ctDNA and the corresponding tumor tissue. Tumor heterogeneity comes from three aspects: intratumor, intertumor, and temporal heterogeneity. Tissue only represents the mutation information of the tumor tissue site, while ctDNA represents the mutation information of all tumor tissues [6, 24]. The preprocessing and testing methods of blood samples will also affect the test results. Our pooled results found that there is a big difference in sensitivity between plasma and serum-derived ctDNA [0.76 (0.73-0.78) vs 0.47 (0.38-0.56)] but are basically the same in specificity [0.94 (0.91-0.96) vs 0.94 (0.86-0.98)]. At present, the ctDNA extraction efficiency of different extraction kits vary greatly, and there is no uniform quality judgment standard between different extraction methods [25, 26]. In the investigation of detection methods, we found that BEAMing (0.78, 95% CI: 0.74-0.81) has a higher sensitivity than Allele-specific PCR (0.60, 95% CI: 0.54-0.65). In addition, we also found that ctDNA has a higher sensitivity in the diagnosis of melanoma patients in North America [0.76 (0.73-0.79) vs. 0.63 (0.57-0.69)], while the specificity difference is small [0.94 (0.91-0.97) vs. 0.93 (0.88-0.97)]. The above results indicate that different regions, different sample sources, and different detection methods will have an impact on the diagnostic performance.

There are still the following issues with this study: first, there aren't many research on the reliability of melanoma diagnosis by ctDNA; as a result, the group's literature is smaller and of varying quality. In the future, more studies of higher quality need to be included, and further studies on possible heterogeneity factors will be made. Second, the included literature uses the assessment of the consistency of ctDNA and tissue biopsy results as evaluation indicators, and there is a possibility that the authors prefer to publish positive results. Third, the number of cases included in the enrollment literature is small, which will affect the accuracy of the statistical results.

## 5. Conclusion

In patients with melanoma, the diagnostic outcomes between ctDNA and matched tumor tissues were more reliable, according to our pooled results. ctDNA has the advantages of low trauma, convenient dynamic monitoring, simple operation, etc., and it is expected to become an auxiliary method for the diagnosis of melanoma gene mutations.

## Figures and Tables

**Table 1:** Baseline characteristics of the included studies.

Author	Year	Country	Sample size	TNM staging	Sample source	ctDNA detection method	Mutation alleles	TP	FP	FN	TN	Sensitivity	Specificity
Yancovitz [12]	2007	USA	17	IV	Plasma	Mutant-specific PCR	BRAF	7	2	5	3	58%	60%
Board [13]	2009	USA	94	IV	Serum	Allele-specific PCR	BRAF	25	3	20	46	56%	94%
Pinzani [9]	2010	Italy	46	II-IV	Plasma	Allele-specific PCR	BRAF	45	3	1	15	97%	83%
Aung [14]	2014	UK	108	IV	Serum	Allele-specific PCR	BRAF	31	2	43	32	42%	94%
					Plasma	Allele-specific PCR	BRAF	38	2	36	32	51%	94%
Santiago-Walker [15]	2015	USA	746	III-IV	Plasma	BEAMing	BRAF	504	2	157	83	72%	89%
Janku [16]	2016	USA	36	IV	Plasma	Allele-specific PCR	BRAF	17	2	9	8	65%	80%
Mosko [17]	2016	UK	122	III-IV	Plasma	UltraSEEK	BRAF	53	2	19	48	74%	96%
Rowe [18]	2018	USA	55	IV	Plasma	BEAMing	BRAF	33	0	5	17	87%	100%
Long-Mira [19]	2018	France	19	IV	Plasma	Allele-specific PCR	BRAF	8	1	2	8	80%	89%
Haselmann [20]	2018	USA	187	III-IV	Plasma	BEAMing	BRAF	56	8	9	114	86%	93%

**Table 2:** Subgroup analysis of ctDNA in diagnosing melanoma.

Variables		Number of studies	Sensitivity			Specificity			Pooled PLR	Pooled NLR	Pooled DOR	Pooled AUC
			*I* ^2^ *(%)*	*P* value	Pooled sensitivity	*I* ^2^ *(%)*	*P* value	Pooled specificity				
Country	North America	6	74	0.0018	0.76 (0.73-0.79)	57.9	0.0366	0.94 (0.91-0.97)	8.15 (2.95-22.51)	0.30 (0.19-0.45)	30.39 (8.63-10.7.01)	0.8018
	Europe	5	92.9	≤0.001	0.63 (0.57-0.69)	0.0	0.5482	0.93 (0.88-0.97)	9.07 (5.01-16.43)	0.35 (0.20-0.61)	28.66 (13.33-61.63)	0.9576

Sample source	Plasma	9	83.8	≤0.001	0.76 (0.73-0.78)	46.9	0.0576	0.94 (0.91-0.96)	8.30 (4.08-16.88)	0.27 (0.18-0.40)	40.36 (15.75-103.43)	0.9282
	Serum	2	52.4	0.1474	.0.47 (0.38-0.56)	0.0	0.9639	0.94 (0.86-0.98)	8.12 (3.39-19.47)	0.56 (0.42-0.73)	14.93 (5.57-40.02)	/

ctDNA detection method	Allele-specific PCR	6	90.5	≤0.001	0.60 (0.54-0.65)	0.0	0.6026	0.92 (0.86-0.95)	6.73 (4.00-11.35)	0.45 (0.32-0.64)	18.29 (9.44-35.46)	0.9271
	BEAMing	3	65.8	0.0538	0.78 (0.74-0.81)	46.2	0.1556	0.96 (0.92-0.98)	21.36 (10.40-43.84)	0.18 (0.10-0.33)	117.66 (50.17-275.94)	0.9539

## Data Availability

Data are present in the manuscript.
